# Prevalence and risk factors for cervical intraepithelial neoplasia in HIV-infected women in Salvador, Bahia, Brazil

**DOI:** 10.1590/S1516-31802010000400004

**Published:** 2010-07-01

**Authors:** Paula Matos Oliveira, Rone Peterson Cerqueira Oliveira, Iane Érica Martins Travessa, Marques Vinícius de Castro Gomes, Maria Lícia de Jesus dos Santos, Maria Fernanda Rios Grassi

**Affiliations:** I MD. Doctoral student in the Postgraduate Program on Medicine and Human Health, Escola Bahiana de Medicina e Saúde Pública (EBMSP), Salvador, Bahia, Brazil.; II MD, MSc. Assistant professor, Gynecological Service, Escola Bahiana de Medicina e Saúde Pública (EBMSP), Salvador, Bahia, Brazil.; III Scientific initiation student, Escola Bahiana de Medicina e Saúde Pública (EBMSP), Salvador, Bahia, Brazil.; IV MD. Gynecologist at the AIDS Reference Center of Bahia (Centro Especializado em Diagnóstico, Assistência e Pesquisa, CEDAP), Salvador, Bahia, Brazil.; V MD, PhD. Head of the Advanced Public Health Laboratory, Centro de Pesquisa Gonçalo Muniz (CPqGM), Fundação Oswaldo Cruz (Fiocruz), Salvador, Bahia, Brazil.

**Keywords:** HIV, Cervical intraepithelial neoplasia, Prevalence, Risk factors, Brazil, HIV, Neoplasia intra-epitelial cervical, Prevalência, Fatores de risco, Brasil

## Abstract

**CONTEXT AND OBJECTIVE:**

The human immunodeficiency virus (HIV) is frequently associated with high-grade intraepithelial neoplasia. Immunosuppression and high HIV viral load are the main risk factors for cervical intraepithelial neoplasia (CIN). The aim of this study was to determine the prevalence of CIN in HIV-infected women in Salvador, Bahia, Brazil, and to describe the risk factors in comparison with non-infected women.

**DESIGN AND SETTING:**

Cross-sectional study at the AIDS Reference Center of Bahia and the Gynecological Outpatient Clinic of Fundação Bahiana para o Desenvolvimento da Ciência, in Salvador, Bahia, Brazil.

**METHODS:**

Sixty-four HIV-infected women and 76 uninfected women from Salvador were enrolled between May 2006 and May 2007. Associations between CIN and presence of HIV infection, HIV viral load, proportion of T CD4+ lymphocytes and risk factors were evaluated. The independence of the risk factors was investigated using logistic regression.

**RESULTS:**

CIN was more prevalent among HIV-infected women than in the control group (26.6% versus 6.6%; P = 0.01). The odds ratio for CIN among HIV-infected women was 3.7 (95% confidence interval, CI: 1.23-11; P = 0.01), after adjusting for the following variables: age at first sexual intercourse, number of partners, number of deliveries and previous history of sexually transmitted disease.

**CONCLUSION:**

The prevalence of CIN among HIV-infected women was significantly higher than among women without HIV infection. HIV infection was the most important risk factor associated with the development of cervical lesions.

## RESUMO

**CONTEXTO E OBJETIVO:**

O vírus da imunodeficiência humana (HIV) está frequentemente associado à neoplasia intraepitelial de alto grau. Imunossupressão e carga viral do HIV elevada são os principais fatores de risco para neoplasia intra-epitelial cervical (NIC). O objetivo deste estudo foi determinar a prevalência de NIC em mulheres infectadas pelo HIV, em Salvador, Bahia, Brasil e descrever os fatores de risco, comparando-as com mulheres não infectadas.

**TIPO DE ESTUDO E LOCAL:**

Estudo transversal no Centro de Referência de Aids da Bahia e Ambulatório de Ginecologia da Fundação Bahiana para o Desenvolvimento da Ciência, em Salvador, Bahia, Brasil.

**MÉTODOS:**

Foram incluídas no estudo 64 mulheres infectadas pelo HIV e 76 não infectadas provenientes de Salvador, no período de maio de 2006 a maio de 2007. Foi avaliada a associação entre NIC e presença da infecção pelo HIV, carga viral do HIV, proporção de linfócitos T CD4+ e fatores de risco. A independência dos fatores de risco foi verificada pela regressão logística.

**RESULTADOS:**

A prevalência de NIC foi maior nas mulheres infectadas pelo HIV que no grupo controle (26,6% *versus* 6,6%; P = 0,01). A razão de chances para NIC em mulheres infectadas pelo HIV foi 3,7 (95% intervalo de confiança, IC: 1,23-11; P = 0,01) após ajuste das variáveis: idade da primeira relação sexual, número de parceiros, número de partos e história prévia de doença sexualmente transmissível.

**CONCLUSÃO:**

A prevalência de NIC foi significativamente maior em mulheres infectadas pelo HIV que naquelas não infectadas. A infecção pelo HIV foi o fator de risco mais importante associado com o desenvolvimento de lesões cervicais.

## INTRODUCTION

It is estimated that over one million women worldwide currently have cervical cancer, mostly undiagnosed. Over the past three decades, cervical cancer rates have fallen in most of the developed world, probably as a result of screening and treatment programs. In contrast, rates in developing countries have risen or remained unchanged. Each year, at least 274,000 women die from invasive cervical cancer, mainly in developing countries, where access to screening services is limited.^1^

Brazil has a continental size and marked regional inequalities. Cervical cancer is the third most common cancer among women in Brazil. In the state of Bahia, in the northeastern region of the country, the estimated risk is 13.55 cases per 100,000 women.^2^ Infection by human papillomavirus (HPV) is the main cause of cervical intraepithelial neoplasia (CIN), which is a precursor lesion for cervical cancer.^3^ In addition to HPV infection, the presence of cofactors such as young age at first sexual intercourse, large number of sexual partners and high-risk sexual behavior of the partner significantly increase the risk of CIN.^3^ Long-term use of oral contraceptives, high parity and smoking are also established factors for the development of CIN and cervical cancer among HPV-infected women.^3,4^

It is well documented that women infected by the human immunodeficiency virus (HIV) have higher prevalence of HPV infection and CIN of the uterine cervix. HIV infection is frequently associated with higher grade cervical dysplasia. Among such patients, these lesions have a worse outcome and progress faster than in immunocompetent patients. Such lesions are difficult to treat, with a high recurrence rate. However, it remains unclear whether immune depletion is the only active mechanism connected with HIV infection and CIN.^5,6^

There are few studies on the prevalence of CIN among HIV-infected women in Brazil, especially in Salvador, Bahia, a state that has sociodemographic characteristics similar to those of African countries.

## OBJECTIVE

The aim of this study was to report on the prevalence of cervical cytological abnormalities among HIV-infected women and to describe the risk factors associated with CIN in this group in Salvador, Bahia.

## MATERIAL and Methods

### Study population and procedure

Sixty-four HIV-infected women who were referred to the AIDS Reference Center of Bahia (Centro Especializado em Diagnóstico, Assistência e Pesquisa, CEDAP) and 76 women without HIV infection who visited the Gynecological Outpatient Clinic of the Bahia Foundation for Science Development (Fundação Bahiana para o Desenvolvimento da Ciência), in Salvador, Bahia, Brazil, between May 2006 and May 2007, were included in this study. The patients were invited to participate in the study when they came for a routine visit.

The inclusion criteria were that the women should be older than 18 years of age, sexually active and serologically positive for HIV (for the HIV group) or negative (for the control group). The exclusion criteria were pregnancy or postpartum status, use of vaginally applied medication over the three days prior to cytological sample collection, sexual intercourse or recent douching over the 48 hours preceding the examination, or vaginal bleeding.

The study was approved by the committee for the protection of human subjects of the Gonçalo Moniz Research Center (Centro de Pesquisa Gonçalo Muniz, CPqGM), Oswaldo Cruz Foundation (Fundação Instituto Oswaldo Cruz, FIOCRUZ), Bahia. All patients signed an informed consent form prior to admission.

### Specimen collection

Specimens for Papanicolaou smears were collected from the ectocervix and endocervix using an Ayres spatula and cytobrush, respectively. Squamous cell abnormalities seen in the Papanicolaou smears were classified as low-grade or high-grade squamous intraepithelial lesions, in accordance with the Bethesda System.^7^ Colposcopic examinations were performed on all of the women by the gynecologist. If a lesion was indicated by the colposcopy or cytology results, it was further evaluated by means of biopsies, which were examined and classified in accordance with the CIN system.^8^

Prior to enrollment, all the women were properly tested for HIV. All the T CD4+ lymphocyte counts and the viral load values were obtained from medical records when the tests were carried out not more than six months prior to the visit. The T CD4+ lymphocyte counts were determined by means of flow cytometry and the HIV viral load by means of the polymerase chain reaction (PCR). Standardized demographic and clinical data were obtained by means of specific questionnaires.

### Statistical analysis

This was an analytical cross-sectional study with a control group. The cytological and histological samples from the HIV-infected women were compared with those of the control group, using t-tests for continuous variables and chi-square tests or Fisher's exact test for categorical variables. We examined the association between CIN and the presence of HIV and immunosuppressive status (T CD4+ lymphocytes < 500 cells/mm^3^), along with risk factors for CIN. Unadjusted odds ratios (ORs) were calculated to screen for inclusion in an initial multivariate model. Variables that exhibited at least a moderate association (P = 0.10) with the outcome in the presence of these design variables were considered for inclusion in the final models. The statistical analysis was performed using the SPSS software (Statistical Package for the Social Sciences), version 13.0.

## RESULTS

The patients’ mean ages were 30.4 ± 5.5 years for the HIV-infected women and 28.6 ± 5.9 for the uninfected patients. In the HIV group, 34 (56.3%) were treated with highly active antiretroviral therapy (HAART). The mean T CD4+ lymphocyte count was 644 ± 514 cells/mm^3^ and HIV viral load was 3.9 ± 4.3 log_10_ copies/ml (Table 1).

**Table 1.** Demographic characteristics and risk factors for cervical intraepithelial neoplasia (CIN) among HIV-infected women and uninfected women

	Uninfected group		HIV-infected group		P
	n = 76		n = 64		
	n		n		
Age (years) (mean ± SD)	76	28.7 ± 6	64	30.4 ± 6	0.07
Marital status (n, %)					
Married/cohabiting	47	61.8	34	53.1	0.29
Unmarried/non-cohabiting	29	38.2	30	46.9	0.29
Education (n, %)					
< 8 years	26	34.2	40	62.5	0.001
> 8 years	50	65.8	24	37.5	0.001
Family income (n, %)					
< $ 240/month	34	44.7	48	75	< 0.001
≥ $ 240/month	42	55.3	16	25	< 0.001
Risk factors for CIN					
Age at first intercourse (mean ± SD)	76	17.5 ± 3.9	64	16.2 ± 4.0	0.05
Number of partners (mean ± SD)	76	3.6 ± 4.3	64	8.3 ± 13.4	< 0.01
STD history (n, %)	10	13.2	26	40.6 %	< 0.01
No. of deliveries (mean ± SD)	76	1.3 ± 1.2	64	1.7 ± 1.2	0.02
Smokers (n, %)	6	7.9	8	12.5	0.36
HAART users	-	-	38	59.4%	NA
T CD4+ count (mean, SD)	-	-	64	644 ± 551	NA
Viral load (mean, SD)	-	-	64	3.9 ± 4.3	NA

SD = standard deviation; NA = not applicable; HAART = highly active antiretroviral therapy; CD4: cells/mm³; Viral load: log_10_ copies/ml; STD = sexually transmitted disease; P < 0.05 from chi-square test or Student's t test.

The cytological smears differed significantly between the groups: squamous intraepithelial lesions (SIL) were more prevalent in HIV-infected patients (16 out of 64) then in the women without HIV infection (6 out of 76) (p = 0.01) (Table 2). CIN was found in 17 patients (26.6%) in the HIV-infected group and in five uninfected women (6.6%) (p = 0.01) (Table 3). Disagreement between cytology and histology was observed in relation to one HIV-infected patient who had normal cytology but presented CIN1 in the histological test, and in relation to one patient in the control group who had low-grade squamous intraepithelial lesion (LSIL), but her histology was normal.

**Table 2.** Cytological smears from HIV-infected women and uninfected women

	Uninfected group n = 76		HIV-infected group n = 64		
	n	%	n	%	
Normal	26	34.2	10	15.6	P = 0.01
Inflammatory	44	57.9	38	59.4	
LSIL	5	6.6	12	18.8	
HSIL	1	1.3	4	6.2	

LSIL = low-grade intraepithelial lesion; HSIL = high-grade intraepithelial lesion. P < 0.05 from chi-square test or Fisher's test.

**Table 3.** Histology results among HIV-infected women and uninfected women

	Uninfected group n = 76		HIV-infected group n = 64		
	n	%	n	%	
CIN1	2	2.6	11	17.2	P = 0.01
CIN2	2	2.6	5	7.8	
CIN3	1	1.3	1	1.6	

CIN = cervical intraepithelial cervical. P < 0.05 from chi-square test or Fisher's test.

Among the HIV-infected women, the risk factors associated with CIN were young age at first sexual intercourse, large mean number of sexual partners, history of previous sexual transmitted diseases and high number of deliveries (Table 1).

The odds ratio value for CIN among HIV-infected women was 3.7 (95% confidence interval, CI: 1.23-11; p = 0.01) after adjusting for the following variables: age at first sexual intercourse, number of partners, number of deliveries and history of sexually transmitted diseases (Table 4).

**Table 4.** Adjusted odds ratio for risk of developing cervical intraepithelial neoplasia (CIN)

Variables	Adjusted OR	95% CI	P
HIV infection	3.7	1.23-11	0.01
First intercourse	1.0	0.89-1.13	0.9
Number of partners	1.0	0.98-1.07	0.26
History of sexually transmitted diseases	1.4	0.5-4.02	0.51
Number of deliveries	0.7	0.49-1.13	0.17

Adjusted variables: HIV infection, first sexual intercourse, number of sexual partners, STD history, number of deliveries. OR = odds ratio. P < 0.05. CI = confidence interval. Logistic regression.

In the group of HIV-positive women who underwent antiretroviral therapy, the frequency of CIN was 18.4 %, while it was 34.6% in the untreated group, without statistical significance (p = 0.14). Moreover, when the HIV-infected patients were stratified according the T CD4+ cell count, there were no significant differences in the CIN frequencies (Table 5).

**Table 5.** Frequencies of histological findings among HIV-infected women stratified according to T CD4+ lymphocyte counts

T CD4+ lymphocytes	≤ 200 cells/mm³		201-499 cells/mm³		≥ 500 cells/mm³		
	n = 3		n = 27		n = 34		
Histological findings	n	%	n	%	n	%	
CIN1	-	-	3	11.1	8	23.5	P = 0.6
CIN2	-	-	3	11.1	2	5.9	
CIN3	-	-	1	3.7	-	-	

CIN = cervical intra-epithelial neoplasia; SD = standard deviation. P < 0.05 from chi-square test or Fisher's test.

## DISCUSSION

Cervical and vaginal intraepithelial neoplasia occur frequently in immunodeficient women, especially those infected by HIV.^9,10^ Recurrence of CIN2 and CIN3 following treatment consisting of large-loop excision of the transformation zone can reach up to 26% among HIV-infected women, compared with 0.6% among women without HIV infection.^11^ This study confirmed that the prevalence of CIN was higher among HIV-infected women than among uninfected women in Salvador, Bahia, Brazil. The power of the sample size in this study was 84% and the alpha error was 0.05. Odds ratios (OR) with 95% confidence interval (95% CI) were used to evaluate the association between HIV infection and CIN. The outcome variable used for this calculation was the presence of cervical neoplasia intraepithelial. One in four (26.6%) of the HIV-infected women screened presented evidence of CIN. This proportion was similar to that described by Levi et al., who found CIN in 18% of their sample of HIV-infected women in São Paulo, Brazil.^5^ In another cross-sectional study carried out in the city of Vitória, Brazil, the prevalence of HPV infection among HIV-infected women (56.3%) was higher than in uninfected controls (40.7%). Nevertheless, the prevalence of high-grade SIL was low (0.7%), and there was no difference between HIV-infected women and uninfected women. It was suggested that the low prevalence of high-grade SIL might be due to earlier access to healthcare and prompt diagnosis, thereby avoiding occurrences of high-grade SILs.^12^

Several cross-sectional and prospective cohort studies have identified some risk determinants for CIN, including large number of sexual partners (lifetime and recent), young age at first sexual intercourse, smoking, oral contraceptive use, presence of other sexually transmitted diseases (STDs), chronic inflammation, immunosuppressive conditions such as HIV infection and high parity.^13-18^ The presence of high-risk HPV viral load may also be important in predicting high-grade CIN among women with atypical squamous cells or LSIL in their cervical smears.^19^ In the presence of high-risk HPV subtypes, cytological abnormalities may be present in up to 44% of HIV-infected women.^20^ In the present study, the HIV-infected women had their first sexual intercourse at an earlier age, a higher number of sexual partners and a higher prevalence of STDs. Nevertheless, in the multivariate analysis, HIV infection remained independently associated with CIN. Even in the absence of HIV infection, Silva et al., in Pernambuco, also described earlier age of first sexual intercourse, HPV type and smoking as risk factors for CIN.^4^ Parham et al. found that among HIV-infected women, age, CD4+ cell count, and presence of any high-risk HPV type were significantly associated with abnormal cytological smears. In a multivariable logistic regression model, they suggested that the presence of high-risk HPV type was an independent predictor for abnormal cytology (adjusted OR: 12.4; 95% CI: 2.62-58.1; P = 0.02).^21^

The association between CIN severity and HIV infection has been clearly demonstrated.^6,16,17^ Moreover, the impairment of cell immune response observed during HIV infection is associated with inadequate clearance of HPV infection, which is one of the major etiological agents for CIN. In such patients, persistence of HPV infection is common, and infection by multiple HPV subtypes and spontaneous regression of low-grade lesions are rare.^18^

The impact of highly active antiretroviral therapy (HAART) on the prognosis for SIL in HIV-infected women has been analyzed. Antiretroviral treatment reduces the risk of recurrence of cervical lesions, probably by restoring or preserving immune function.^19,20^ Levi et al. observed that 31% of the patients with fewer than 200 cells/μl had abnormal cervical smears, in contrast with 13% of those with counts higher than 200 cells/μl.^5^ In the present study, no relationship was observed between immunosuppression and lesion severity. The T CD4+ lymphocyte count was not statistically different between patients without CIN and patients with low or high-grade neoplasia. This finding is in accordance with other studies that did not find any association between the T CD4+ lymphocyte count and the severity of CIN.^22,23^ Nevertheless, the majority of the HIV-infected patients enrolled in the present study were being treated with HAART and their cell immune response was preserved, with T CD4+ cell counts higher than 500 cells/mm^3^.

However, the immune response against HPV also depends on innate immunity, including macrophages, natural killer cells and cytokine production, which may also be impaired during HIV infection.^24^ Therefore, local cervical immunity, especially with regard to reduced numbers or function of dendritic cells, could explain the progression of cervical neoplasia.^25^

## CONCLUSIONS

In summary, the prevalence of CIN in HIV-infected women was significantly higher than in women without HIV infection. The presence of HIV infection was the most important risk factor associated with the development of cervical lesions, probably because HIV patients are exposed to several risk factors associated with CIN. To prevent the lesions from progressing to invasive cancer, gynecological evaluation, cervical cytological tests and colposcopy should be considered to be essential examinations for HIV-infected woman, even in the presence of higher T CD4+ cell counts. HIV-infected women should be prioritized in HPV screening programs.
